# Case-finding of chronic obstructive pulmonary disease with questionnaire, peak flow measurements and spirometry: a cross-sectional study

**DOI:** 10.1186/1756-0500-7-241

**Published:** 2014-04-16

**Authors:** Bassam Mahboub, Ashraf Alzaabi, Joan B Soriano, Laila Salameh, Yousef AL Mutairi, Afzalhussein A Yusufali, Alawi Alsheikh-ali, Wael Almahmeed, John Haughney

**Affiliations:** 1 Department in Rashid Hospital, Pulmonary medicine, Dubai, United Arab Emirates; 2 Respiratory Division at Zayed Military Hospital, Abu Dhabi, United Arab Emirates; 3 IdISPa-FISIB, Hospital Universitari Son Espases, Palma de Mallorca, Spain; 4 Respiratory Care Services, Dubai Health Authority (DHA), Rashid Hospital, Dubai, United Arab Emirates; 5 Internal Medicine and Cardiology, Hatta Hospital, Dubai Health Authority, Dubai, United Arab Emirates; 6 Institute of Cardiac Sciences, Sheikh Khalifa Medical City (SKMC), Abu Dhabi, United Arab Emirates; 7 University of Aberdeen, Aberdeen, UK; 8 Institute for Clinical Research and Health Policy Studies, Tufts Medical Center and Tufts University School of Medicine, Boston, MA, USA

## Abstract

**Background:**

Spirometry is commonly accepted as the gold standard for the diagnosis of COPD*,* but the reality remains that quality assured spirometry is not or cannot be provided universally around the globe. Adding PEF measurement to a screening questionnaire may rule out airflow limitation compatible with COPD rationalizing spirometry testing.

**Methods:**

We conducted a cross-sectional survey in a sample of individuals 40–80 yrs. old in Dubai, UAE. They were invited to answer a short socio-demographic questionnaire including a report on current, past history of smoking, and had PEF measured, then they conducted spirometry to identify airflow limitation compatible with COPD.

**Results:**

Overall, 525 (91.0%) participants performed PEF and spirometry (68% male, with a mean age of 59 years, 17% UAE Nationals), 24% reported smoking of different sorts. Overall, 68 participants (12.9%, 95% C.I. 10.3% to 16.1%) had airflow limitation compatible with COPD. PEFR alone identified 141participants with airflow limitation compatible with COPD, with specificity of 80% and sensitivity of 73.5%.

**Conclusions:**

PEFR could be an easy, cheap, and non-biased tool to assist with the case-finding of COPD before confirmation with spirometry.

## Background

Undiagnosed airflow limitation (airway obstruction) is common in the general population and is associated with impaired health and functional status [1]. Chronic obstructive pulmonary disease (COPD) is a leading cause of chronic morbidity and mortality worldwide [2]. According to the Global Burden of Disease Study in 2010, COPD was the sixth leading cause of death worldwide in 2001, but moved to third in 2010, just behind ischemic heart disease and stroke [2]. Furthermore, COPD prevalence is greatly underestimated, since it is usually not diagnosed until it is clinically apparent and moderately advanced. It is currently estimated there are 328 million people with COPD in the World [3].

In the Middle East & North Africa (MENA) region, epidemiological data on COPD are scarce. The BREATHE study, a recently conducted international survey in 2012, described the prevalence of symptoms that could be COPD-related in each MENA country. The lowest age- and gender-adjusted prevalence was in the United Arab Emirates (UAE) with 1.9% of participants (95% CI 1.4 to 2.4) [4]. In another study conducted in 2010 by Alzaabi et.al., COPD prevalence in those 40–80 years old in Abu Dhabi, UAE was also low with 3.7% (95% CI 2.0 to 5.3) [5].

Earlier COPD diagnosis should produce substantial individual health and Public Health benefits [6,7] and therefore there is an pressing need for case-finding strategies to support the early diagnosis of COPD. Case finding can be active (e.g. targeting smokers over 40 years of age with case-finding questionnaires) or passive (e.g. waiting for people to go to the doctor) [8,9].

Spirometry is commonly accepted as the gold standard for the diagnosis of COPD [10,11]. The American Association for Respiratory Care supports the National Lung Health Education Program (NLHEP) to promote the appropriate use of spirometry by primary health care practitioners for the detection of COPD in adult smokers [12]. The reality remains that quality assured spirometry cannot be provided universally around the globe. Another problem that spirometers may not be available or used properly in primary health care due to financial limitations or limited availability of expert technicians or clinicians to perform the procedure [7].

A workshop organized by the US National Institutes of Health (National Heart, Lung, and Blood Institute Division of Lung Diseases (DLD)) identified an urgent need to develop and test a strategy for active case finding of COPD for those who have clinically significant COPD (specifically those with a forced expiratory volume in 1 s (FEV1) ≤ 60% predicted). They suggested this should include a combination of initial risk assessment (via a questionnaire) followed by a simple measurement of peak expiratory flow (PEF) and, as appropriate, full diagnostic pre-bronchodilator_(BD) and post-BD spirometry [13].

Validated questionnaires facilitate early recognition and diagnosis of COPD. Perez-Padilla et al., concluded that adding PEF measurement to a screening questionnaire may rule out severe to very severe COPD without the need for pre- and post-BD spirometry testing. In a recent multicenter study, Burden of Obstructive Lung disease (BOLD), Jithoo and colleagues concluded that the use of peak expiratory flow (PEF), with a 2.2 L.^s-1^.m^-2^ threshold was a simple, cost effective initial screening tool for conducting COPD case-finding in adults aged ≥40 years [7]. Our study aimed to assess the value of PEFR as a screening tool by comparing it to spirometry.

## Methods

We conducted a cross sectional study in Dubai, UAE, in collaboration with the Emirates Cardiac Society during the World Heart Day campaign in September 2012. Participants were recruited from five primary health care centers during routine clinic visits, from two large shopping malls in Dubai, and from Dubai Industrial city, (a large, varied workplace) aiming to represent a valid sampling frame of the local population. This study was approved by the Dubai Health Authority (DHA) research and ethics committee.

### Study population

Adult participants 18 years and older were offered screening for medical conditions with measurements of height, weight, blood pressure, Hemoglobin A1c (Hb A1c), lipid profile, and carbon monoxide (CO). Overall, a total of 1,607 people were screened for cardiopulmonary risk factors and participants 40 years and older were invited for COPD screening including of a short socio-demographic questionnaire, PEFR, and spirometry.

### Questionnaire

**Figure 1:**
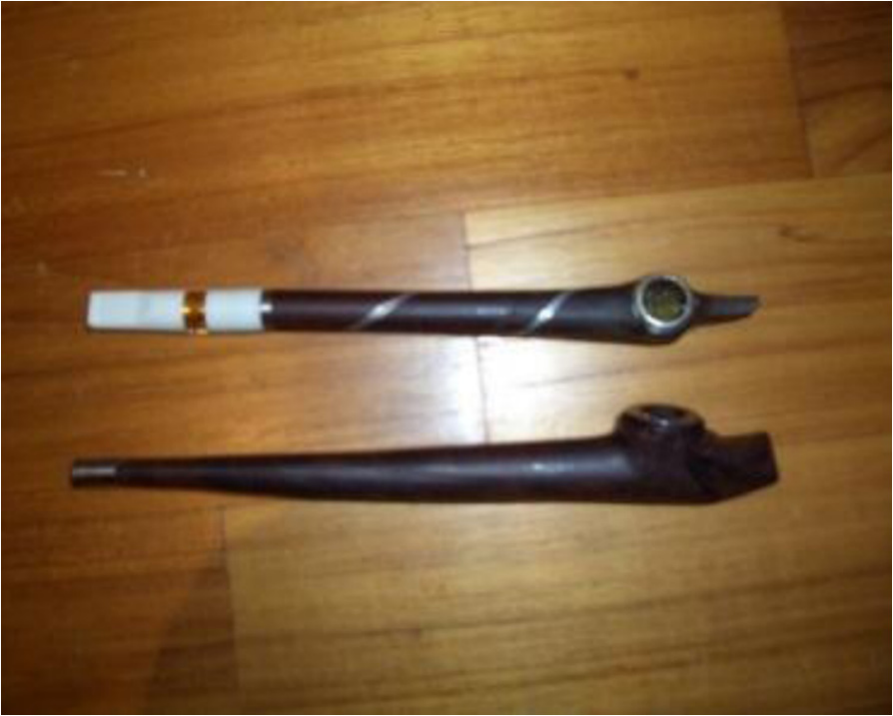
Midwakh.

Data were collected via face-to-face interview, and included data about current and past smoking cigarettes and other local products like Midwakh (a small pipe for smoking tobacco of Arabian origin, (Figure 1) which was traditionally smoked by Bedouins and sailors in the UAE, water pipe and other. We also assessed exposure to domestic biomass and coal pollution (cooking/heating), and occupational exposure to dust [14].

### Peak expiratory flow rate (PEFR)

We measured peak expiratory flow rate (PEFR) in all participants. To correlate with height, PEFR measurements were divided by height squared (m^2^) and expressed in units of L.s^-1^.m^-2.^[7].

### Spirometry

Spirometry was performed by using a handheld spirometer (Vitalograph alpha and one alpha, Vitalograph Ltd UK). The accuracy of the devices were calibrated against our laboratory spirometer (Master screen Care Fusion Corporation, Delaware, USA). Pre-bronchodilator (pre-BD) spirometry was performed to identify airflow limitation (ratio of FEV_1_/FVC < 0.70) compatible with COPD. The severity of airflow limitation was staged according to the Global Initiative for Chronic Obstructive Lung Disease (GOLD) guidelines as mild, moderate, severe, and very severe according to FEV_1_ (% predicted) as >80%, 50-79%, 30-49%, or < 30% respectively.

#### Statistical methods

Data was reviewed by a central committee, and values that were considered as potential errors or outliers were individually addressed. Comprehensive tabulations with ranges, mean and standard deviation of all predefined quantitative variables, and percentages of all predefined qualitative variables, were conducted.

The results for each variable are shown as the mean with standard deviation in the case of continuous variables, and the number of cases for each category and frequency regarding the total number of responses in the case of categorical variables. The prevalence of airflow limitation and its 95% confidence interval in total and by subgroups were calculated. The statistical significance of variables was assessed first with ANOVA and then a bilateral test for continuous variables, and a Chi-squared test for categorical variables. In a final logistic multivariate analysis, the reference categories were: age between 40 – 49 years; female; never smoker; and University degree education. In all analyses, a P value below 0.05 was considered to be statistically significant.

## Results

**Figure 2:**
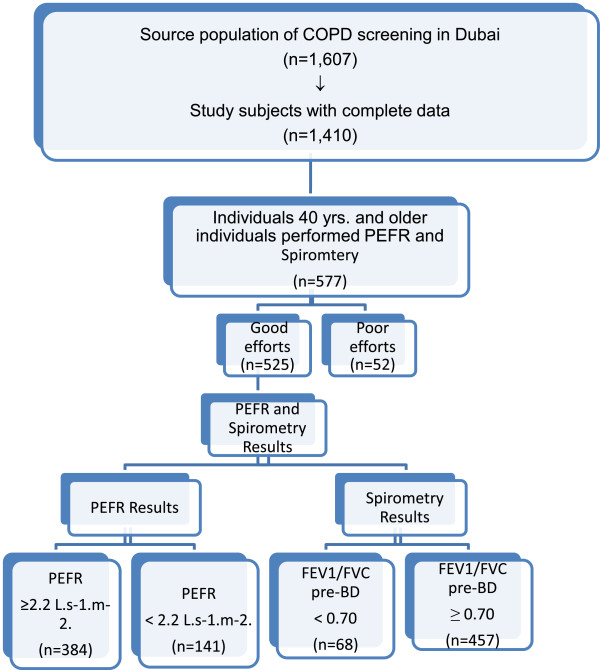
STROBE flow-chart of participants.

A STROBE flow-chart of participants is presented in (Figure 2), indicating the sequential recruitment of individuals. As stated, 1,607 individuals participated from the five primary health care centers, the two shopping malls, and Dubai Industrial City. Of them, 1,410 participants had a minimum data set, and of these577 participants were 40 years and older. These 577 participants were invited to perform PEFR, then spirometry. Overall, 525 (91.0%) conducted quality controlled pre-BD spirometry. Participants were 68% male, with a mean age of 59 years, 17% were UAE Nationals, and 24% reported smoking of some sorts.

Table 1 shows the numbers of participants who might have airflow limitation compatible with COPD by using PEFR measurements (using proposed method PEFR **(L**^**.s-1**^**.m**^**-2**^**)** (≤1.3, >1.3–1.8, >1.8–2.2, >2.2 L.s-1.m-2).

**Table 1:** Participants PEFR results according proposed method PEF per m2

**Pre-BD PEF per Ht**^**2 **^**(L**^**.s-1**^**.m**^**-2**^**) results**	**N=**	**%**
≤1.3	40	7.6%
>1.3–1.8	44	8.3%
>1.8–2.2	57	10.6%
>2.2	384	73.1%

**Figure 3:**
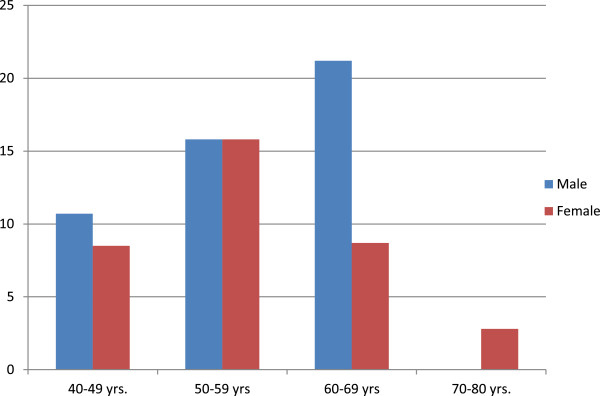
Prevalence of COPD (and 95% C.I.) by gender and age group.

By using spirometry overall 68 participants (12.9%, 95% C.I. 10.3% to 16.1%) had airflow limitation compatible with COPD. Figure 3 shows the prevalence of COPD by gender and age group.

Participants who had airflow limitation compatible with COPD were older than those who had normal lung function (mean ± SD) 52 ± 8.8 years vs. 49.2 ± 7.7 years, p = 0.006). A comparison of the demographic characteristics of participants, smoking prevalence, exposure to domestic biomass, and occupational exposure to dust between participants who had airflow limitation compatible with COPD and those with normal lung function is shown in Table 2. Occupational exposure to dust was associated with airflow limitation compatible with COPD (p = 0.006).

**Table 2:** Demographic characteristics of participants

**Variable**	**COPD (n = 68)**	**Normal lung function (n = 457)**	**P**
Male gender, n (%)	47 (69.1%)	311 (68.1%)	1.000
Age in years, mean ± SD	52 (±SD 8.8)	49.2 (SD 7.7)	0.006
Nationality, n (%)			0.488
UAE	9 (13.2%)	79 (17.3%)	
Other	59 (86.8%)	378 (82.7%)	
Height in cm, mean ± SD	164.9 (±SD 8.6)	164.82 (±SD 8.7)	0.890
Weight in kg, mean ± SD	76.3 (±SD 12.9)	77 (±SD15.4)	0.737
BMI in kg/m^2^, mean ± SD	28.4 (±SD 4.6)	28.4 (±SD 4.7)	0.902
Education, n (%)			0.397
Primary	34 (50.0%)	231 (50.5%)	
Diploma	3 (4.4%)	24 (5.3%)	
College	30 (44.1%)	174 (38.1%)	
Higher Education	1 (1.5%)	28 (6.1%)	
Any smoking, n (%)	17 (25.0%)	111 (24.3%)	0.881
Cigarette smoking			
Smokers, n (%)	18 (26.4%)	115 (25.2%)	1.000
Number of cigarettes/day, mean ± SD	14.4(±SD 11)	12 (±SD 10.4)	0.493
Midwakh smokers, n (%)	0 (0.0%)	1 (0.3%)	1.000
Water pipe smoking			
Smokers, n (%)	1 (3.4%)	13 (3.9%)	1.000
Heads of waterpipe/month, mean ± SD	4.1 (±SD 22.3)	1.1 (±SD 7.5)	0.098
Other Smoking			
Smokers, n (%)	1 (3.4%)	3 (0.9%)	0.294
Other tobacco per week, mean ± SD	0.9 (±SD 5.1)	0.2 (±SD 1.9)	0.082
Exposure to domestic biomass and coal pollution (cooking/heating), n (%)	12 (17.6%)	52 (11.4%)	0.162
Occupational exposure to dust, n (%)	26 (38.2%)	102 (22.3%)	0.006
Hospital admissions due to pulmonary problems in childhood, n (%)	4 (5.9%)	20 (4.4%)	0.536

In (Table 3) we present the crude and the adjusted risks for COPD (OR and 95% confidence interval). In bivariate analysis, only increasing age and previous occupational exposure to dust were associated with COPD (both p < 0.05). In the multivariate analysis, there was a trend for older, non-UAE nationals, less educated, and smokers to have high risk of COPD, but again only occupational exposure to dust remained statistically significant.

**Table 3:** Crude and adjusted risk of COPD (OR and 95% confidence interval)

**Variable**	**Bivariate**	**Adjusted**
Male gender	1.05 (0.61-1.89)	0.93 (0.41-1.70)
Age in years		
40-49 yrs.	1	1
50-59 yrs.	1.67 (0.95-2.93)	1.66 (0.94-2.95)
60-69 yrs.	1.70 (0.76-3.83)	1.60 (0.70-3.66)
70-80 yrs.	3.34 (0.84-13.28)	4.36 (1.03-18.48)
UAE National	0.76 (0.39-1.43)	0.68 (0.30-1.53)
Education		
Primary	1	1
Diploma	0.85 (0.24-2.97)	0.89 (0.25-3.19)
College	1.17 (0.69-1.99)	1.15 (0.66-2.02)
Higher Education	0.24 (0.03-1.84)	0.27 (0.03-2.69)
Smoking any	1.04 (0.58-1.87)	1.10 (0.59-2.06)
Cigarette user	1.05 (0.57-1.94)	
Midwakh user	0.92 (0.84-0.95)	
Water pipe user	0.87 (0.11-6.9)	
Other user	3.77 (0.38-37.40)	
Biomass exposure	1.67 (0.84-3.32)	
Occupational exposure	2.16 (1.26-3.68)	2.07 (1.20-3.59)
Respiratory infection in childhood	1.37 (0.45-4.12)	

Participants who had airflow limitation compatible with COPD were categorized by severity as 33.3% mild (GOLD stage I) 55.5% moderate (GOLD stage II) 11.1% severe (GOLD stage III), and none with very severe COPD (GOLD stage IV) as showed below in (Table 4).

**Table 4:** Clinical characteristics of participants

**Variable**	**COPD (n = 68)**	**Normal lung function (n = 457)**	**p**
FEV_1_ pre-BD, mean ± SD	1.9 (±SD 0.6)	2.7 (SD 0.6)	< 0.001
FVC pre-BD, mean ± SD	3.2 (±SD 1.0)	3.1 (SD 1.0)	0.700
FEV_1_/FVC pre-BD, mean ± SD	0.60 (±SD 0.09)	0.86 (SD 0.09)	< 0.001
PEFR pre-BD, mean ± SD	208.9 (±SD 146.0)	502.1 (SD 220.7)	< 0.001
Severity of airflow limitation, %			
Mild	33.3%	-	-
Moderate	55.5%		
Severe	11.1%		
Very Severe	0.0%		

We found that out of 68 participants with COPD in our study, 50 participants would have been identified as COPD by using a proposed threshold of PEFR of 2.2 L.^s-1.^m^-2^; Hence PEF measurements resulted in 73.5% sensitivity and 80% specificity for the diagnosis of COPD.

In (Table 5) there were no clinically or statistically significant differences observed by gender, age, smoking status or height in COPD patients as diagnosed by PEFR.

**Table 5:** Usefulness of peak expiratory flow rate (PEFR) for COPD screening

**Variable**	**PEFR < 2.2 L.**^**s-1**^**.m**^**-2 **^**(n = 50)**	**PEFR ≥ 2.2 L.**^**s-1**^**.m**^**-2 **^**(n = 18)**	**P**
Male gender, n (%)	33 (66.0%)	13 (76.5%)	0.550
Age in years, mean ± SD	52.7 (SD 9.3)	50.5 (±SD 7.4)	0.393
Smoker, n (%)	12 (24.0%)	5 (29.4%)	0.749
Height in cm, mean ± SD	164.4 (±SD 8.8)	165.2 (±SD 5.6)	0.723

## Discussion

We found that the measurement of pre-BD PEF as a screening tool in adults with a high risk of COPD was able to identify individuals who were most likely to benefit from confirmatory spirometry.

Our results support previous work by Jithoo et al., who suggested using a PEFR threshold of < 2.2 L.^s-1^.m^-2^. There were no differences observed in gender, age, smoking status or height among in people with COPD with PEFR values above or below the threshold emphasizing the value of this approach in COPD case finding. Further research is awaited and issues that need to be addressed have been raised [15].

In our study, PEF measurements resulted in 73.5% of sensitivity and 80% specificity. This is comparable to Jitho’s study (83-84%% sensitivity overall and 91–93% sensitivity for severe COPD). That difference could be due to different type of populations and number of participants. Ours was a single location study in Dubai with 525 participants while Jitho et al., was based on population samples from 14 different centers comprising 10,712 participants around the world [7].

Our study concluded that previous occupational exposure to dust was associated with airflow limitation compatible with COPD. Previous studies [16,17] found an association between environmental exposures, occupational and biomass exposures, and the prevalence/incidence of COPD. UAE is a fast growing country with many new construction sites, which could explain and support our finding of the statistically significant relationship between occupational exposure and COPD, which is worth further exploration in future studies with a larger sample.

We were unable to confirm smoking or biomass exposure findings. However, this factor had been measured using a simple question during the interview. Moreover, due to the relatively low number of subjects with COPD in the study, the power of our study might not have been enough to detect small effects of smoking or biomass exposure.

Although PEF has not been well recognized for COPD diagnosis, it has been proposed as a good indicator of COPD mortality risk in hospital [18] and of quality of life. It has also been explored in the emergency room assessment of COPD exacerbation [19] and with good reliability for home assessment in patients with COPD [20].

### Advantages and limitations

Our study has some strength, including novelty given the scarcity of previous spirometry studies in our environment and the large response rate. However, a main limitation of our study is the use of pre-bronchodilator spirometry for defining airflow limitation compatible with COPD. As a consequence, given that all major COPD guidelines [1,10,15] recommend post-BD spirometry, some subjects with asthma with fully reversible obstruction could have been falsely classified as having COPD leading to overestimation of COPD patients. Other limitations in our study include the following: participants were volunteers and hence a potential selection bias could be present; the relatively small sample size prevented us from conducting further subgroup analysis, although our multivariate analysis appears robust.

## Conclusion

PEFR, with or without a questionnaire, could be used as a simple tool in the Primary health care setting to screen smoker of more than 40 years age for airflow limitation compatible with COPD.

## Competing interest

The authors declare that they have no conflicts of interest in relation to this article.

## Authors’ contributions

All authors contributed to the manuscript and its review. In addition the following functions were conducted. BM: chief investigator and guarantor of study; AAZ: co-investigator. JS: methods advisor, statistical analysis LS: Data processing and medical writing; YAM: field data supervisor; A: logistic support; YA: logistic lead and data processing.
